# Oxaliplatin desensitization for gastrointestinal cancer patients with hypersensitivity reactions: real-world experience from Vietnam

**DOI:** 10.3389/fonc.2026.1876656

**Published:** 2026-09-30

**Authors:** Le Trang Nguyen, Van Thang Nguyen, Hong Thach Nong, Nguyen Thanh Dat Doan, Hoang Tuan La, Thi Mai Vu, Thanh Thien Le, Thanh Dung Quach, Quynh Lan Phan, Van Dinh Nguyen, Ngoc May Le

**Affiliations:** 1 Pharmacy Department, Vinmec Times City International Hospital, Hanoi, Vietnam; 2 College of Health Sciences, VinUniversity, Hanoi, Vietnam; 3 Hanoi University of Pharmacy, Hanoi, Vietnam; 4 Allergy and Immunology Department, Vinmec Times City International Hospital, Hanoi, Vietnam; 5 Vinmec-VinUni Institute of Immunology, College of Health Sciences, VinUniversity, Hanoi, Vietnam; 6 Center for Innovations in Health Sciences, VinUniversity, Hanoi, Vietnam; 7 Vinmec Healthcare System, Hanoi, Vietnam; 8 Oncology Department, Vinmec Times City International Hospital, Hanoi, Vietnam

**Keywords:** chemotherapy - oncology, desensitization therapy, FOLFOX, gastrointestinal cancer, hypersensitivity reaction, oxaliplatin (43805), skin test (medline id d012882)

## Abstract

**Introduction:**

Oxaliplatin-induced hypersensitivity reactions (HSRs) can interrupt clinically indicated treatment for gastrointestinal malignancies. We evaluated the early implementation of a multidisciplinary oxaliplatin desensitization pathway at Vinmec Times City International Hospital.

**Methods:**

We conducted a retrospective case series of patients who underwent oxaliplatin desensitization between 2022 and 2025 following an index HSR. Clinical characteristics, skin testing results, desensitization procedures, breakthrough reactions (BTRs), and subsequent oxaliplatin treatment completion were reviewed.

**Results:**

Ten patients underwent desensitization after index HSRs occurring at a median of six oxaliplatin cycles (range, 2–11); six index reactions were Brown grade 2. Skin prick testing was negative in all patients, whereas intradermal testing was positive in six, negative in three, and equivocal in one. Three patients initially underwent a three-bag desensitization procedure and seven a four-bag procedure. Eight BTRs occurred in six patients, none requiring epinephrine. Seven patients completed all additional oxaliplatin cycles planned by their oncology team, receiving a median of four additional cycles. Three patients discontinued oxaliplatin after BTRs: two because of recurrent reactions despite procedure modification and one because the patient declined further treatment after a single BTR in the metastatic setting.

**Discussion:**

Multidisciplinary oxaliplatin desensitization enabled most patients in this early case series to continue and complete planned oxaliplatin treatment, although BTRs were common. Given the small, retrospective cohort, these findings are descriptive and require further study to establish comparative safety or efficacy, superiority of a specific desensitization procedure, or predictors of treatment completion.

## Introduction

Oxaliplatin is a third-generation platinum-based chemotherapeutic agent with alkylating-like properties. Following aquation, it forms reactive platinum complexes that induce DNA crosslinks, thereby inhibiting DNA replication and transcription and producing cell-cycle–nonspecific cytotoxic effects (1). Oxaliplatin plays a central role in the treatment of gastrointestinal malignancies, particularly colorectal and gastric cancers, across both adjuvant and metastasis settings. It is a core component of standard chemotherapy regimens, including FOLFOX, CAPOX, and FOLFOXIRI, and may also be administered in combination with targeted or immune-based therapies in selected clinical settings (2–4).

The clinical use of oxaliplatin may be limited by hypersensitivity reactions (HSRs), which can occur within minutes of administration and during any treatment cycle (1). The reported incidence of oxaliplatin-induced HSRs is approximately 9%–20%, while severe or anaphylactic reactions occur less frequently (1, 5, 6). Oxaliplatin HSRs are clinically heterogeneous. The principal recognized phenotypes include type I reactions, which are often compatible with an IgE-mediated mechanism; cytokine-release reactions, characterized by manifestations such as fever, chills, or rigors; and mixed reactions combining features of both phenotypes (6, 7). Because clinical manifestations may overlap and confirmatory biomarkers are not routinely available, the underlying immunologic endotype cannot always be determined from the clinical presentation alone.

Diagnostic evaluation should therefore integrate a detailed clinical history with available *in vivo* and *in vitro* investigations (6, 7). Skin testing, including skin prick and intradermal testing, may support the evaluation of immediate platinum hypersensitivity, although false-negative results can occur (6, 7). The basophil activation test (BAT) has emerged as a complementary *in vitro* tool for assessing immediate reactions to platinum compounds and may provide additional information regarding basophil reactivity and reaction phenotype (8, 9). However, limited clinical availability, methodological heterogeneity, and the absence of universally standardized drug-specific thresholds currently restrict its routine use (9).

Oxaliplatin HSRs may involve cutaneous, respiratory, cardiovascular, and gastrointestinal manifestations, including flushing, pruritus, urticaria, angioedema, bronchospasm, hypotension, nausea, and abdominal symptoms (1, 6). Respiratory or throat symptoms occurring during oxaliplatin infusion nevertheless require careful interpretation. Acute oxaliplatin-induced pharyngolaryngeal dysesthesia, particularly when triggered or exacerbated by cold exposure, may cause subjective dyspnea, throat tightness, or difficulty swallowing and can mimic an HSR. Distinguishing these conditions requires consideration of objective allergic manifestations, involvement of multiple organ systems, temporal characteristics, possible cold exposure, response to treatment, and the overall allergy assessment (1, 6). Clinically suspected HSRs may require treatment interruption or permanent discontinuation and can prevent patients from receiving an otherwise indicated oxaliplatin-containing regimen.

For selected patients in whom continued oxaliplatin treatment remains clinically important, rapid drug desensitization provides a strategy for temporary reintroduction of the drug under controlled conditions (6). Desensitization protocols administer progressively increasing doses through a series of predefined infusion steps under close clinical monitoring (6, 7). This controlled exposure induces temporary hyporesponsiveness of effector cells in type I reactions and may attenuate mediator release in other immediate reaction phenotypes (6). Desensitization can therefore allow selected patients to receive additional oxaliplatin despite a previous HSR, although breakthrough reactions may still occur and repeated desensitization is required for each subsequent administration. Its implementation requires appropriate patient selection, standardized drug preparation and infusion procedures, close monitoring, emergency preparedness, and coordination among oncology, allergy, and clinical pharmacy teams.

Published experience with oxaliplatin desensitization has predominantly originated from specialized centers with established drug-allergy programs. Evidence describing how such pathways are implemented and operated in Vietnamese oncology practice remains limited. We therefore conducted this retrospective case series to describe the early implementation of a multidisciplinary oxaliplatin desensitization pathway at a Vietnamese cancer center and to report the clinical characteristics, protocol allocation, and patient- and procedure-level outcomes among patients who underwent desensitization after oxaliplatin HSRs.

## Methods

### Data collection

This retrospective case series included all patients at Vinmec Times City International Hospital (Hanoi, Vietnam) who developed a clinically suspected HSR during oxaliplatin treatment and subsequently underwent oxaliplatin desensitization between January 2022 and December 2025. Demographic characteristics, cancer diagnosis and treatment setting, complete oxaliplatin-containing regimen, previous oxaliplatin exposure, cycle number and timing of the index reaction, reaction manifestations and treatment, skin-test findings, desensitization procedures, BTRs, and subsequent treatment course were extracted from the medical records.

Index and breakthrough reactions were graded retrospectively according to the Brown severity classification (12). Grade 1 reactions were limited to cutaneous or subcutaneous manifestations. Grade 2 reactions involved respiratory, cardiovascular, or gastrointestinal manifestations without grade 3 features. Grade 3 reactions were characterized by hypoxia, hypotension, or neurologic compromise. Oncologic response was abstracted when available and categorized according to RECIST version 1.1 (13); these outcomes were reported descriptively and were not attributed to desensitization.

### Allergy evaluation and skin testing

All patients were evaluated by the Allergy and Immunology team before oxaliplatin re-administration. The assessment included review of the index reaction, Brown severity grading, consideration of alternative diagnoses such as oxaliplatin-induced pharyngolaryngeal dysesthesia, and confirmation that continued oxaliplatin treatment remained clinically indicated.

Skin testing consisted of a skin prick test with oxaliplatin at 1 mg/mL, followed, when negative, by an intradermal test at 0.1 mg/mL, based on published protocols (10, 11). Histamine 1 mg/mL and 0.9% sodium chloride were used as positive and negative controls. Tests were read after 15–20 minutes, and a result was considered positive when the wheal was at least 3 mm larger than the negative control. Because higher intradermal concentrations were not tested, negative or equivocal results were interpreted cautiously and did not exclude oxaliplatin hypersensitivity. Serum tryptase, cytokine measurements, and basophil activation testing were not routinely available.

### Desensitization procedures

Desensitization was performed in a monitored oncology infusion unit by allergists, oncologists, oncology nurses, and clinical pharmacists. All patients received diphenhydramine 30 mg, dexamethasone 12 mg, and famotidine 20 mg intravenously approximately 30 minutes before infusion. Vital signs and symptoms were monitored throughout, and emergency medications and resuscitation equipment were immediately available.

The institutional pathway used three-bag or four-bag procedures with sequential oxaliplatin dilutions and stepwise increases in infusion rate, generally at 15-minute intervals. The prescribed dose and final concentration were individualized according to the chemotherapy regimen and body surface area. The institutional oxaliplatin desensitization pathway was first implemented in 2022 and evolved during the 2022–2025 study period as procedures were adapted according to individual patient tolerance and clinical judgment. Representative three-bag and four-bag procedures are presented in **Supplementary Tables S3** and **S4**.

Initial procedure selection was made case by case according to the index-reaction severity and phenotype, skin-test findings, prior oxaliplatin exposure, oncologic indication, and clinical judgment. No prospectively validated allocation algorithm was used. All patients with negative or equivocal intradermal results received an initial four-bag procedure, whereas a positive intradermal result did not determine allocation on its own. Selected patients were de-escalated from four bags to three bags after at least three tolerated procedures, whereas more conservative procedures were used after breakthrough reactions. These decisions describe institutional practice during the implementation period and should not be interpreted as validated risk-stratification or protocol-selection criteria.

### Management of breakthrough reactions

A breakthrough reaction was defined as a new hypersensitivity-compatible reaction occurring during desensitization. When a reaction occurred, the infusion was stopped and the patient was reassessed. Initial treatment consisted of intravenous methylprednisolone 40 mg and diphenhydramine 20 mg. No breakthrough reaction required epinephrine.

After symptom resolution and clinical reassessment, the infusion could be resumed at the previously tolerated step or at a lower rate. Subsequent procedures could be modified by reducing the maximum infusion rate, adding steps, changing from three to four bags, or increasing premedication. No epinephrine was used for breakthrough reactions in this study. Permanent discontinuation was determined according to reaction recurrence, ability to complete the infusion, anticipated oncologic benefit, treatment alternatives, and patient preference. Event-level details are reported in Supplementary **Supplementary Table S2**.

### Outcome definitions

Procedure completion was defined as administration of the intended oxaliplatin dose during an individual desensitization procedure, including procedures resumed after a managed breakthrough reaction. Patient-level treatment completion was defined as completion of all additional oxaliplatin cycles planned by the oncology team. Reasons for permanent noncontinuation were classified as recurrent breakthrough reactions or patient preference after a breakthrough reaction.

### Data analysis

Given the small sample size and descriptive nature of this retrospective case series, no inferential statistical analyses were performed. Continuous variables were summarized using the median and range or interquartile range, as appropriate, and categorical variables were reported as counts and percentages. No formal comparisons, predictive modeling, or estimates of protocol efficacy were performed.

Outcomes were summarized at both patient and procedure levels. Patient-level outcomes included the number of additional oxaliplatin cycles, occurrence of at least one BTR, completion of the treatment planned by the oncology team, and noncontinuation of oxaliplatin after a BTR. Procedure-level outcomes included the total number of desensitization procedures, BTR events, infusion resumption, procedure completion or termination, and modifications before subsequent procedures. Oncologic responses were presented only as descriptive clinical context.

### Ethical considerations

This study was conducted in accordance with the ethical principles outlined in the Declaration of Helsinki and the Good Clinical Practice guidelines of the International Council for Harmonization (ICH-GCP). Research chart abstraction and analysis commenced after approval by the Institutional Review Board of Vinmec International Hospital on March 25, 2026 (Approval No.: 90/2026/CN/HDDD VMEC). The IRB approved retrospective use of clinical records generated during routine care between January 2022 and December 2025 and waived the requirement for individual written informed consent. All data were anonymized before analysis, and no directly identifiable patient information was included in the study dataset or manuscript. No additional interventions were performed for research purposes. All clinical management, including oxaliplatin desensitization procedures, was conducted as part of routine standard care. Data were securely stored and used solely for research purposes.

## Results

### Patient characteristics

Ten patients were included, most of whom were male (**Table 1**). The median age at desensitization was 64 years. Six patients had colon cancer, three had gastric cancer, and one had rectal cancer. Seven patients received oxaliplatin in the metastasis setting and three in the adjuvant setting. Nine index reactions occurred during FOLFOX, comprising 5-fluorouracil, leucovorin, and oxaliplatin, and one occurred during CAPOX.

**Table 1:** Patient characteristics and index reactions.

Patient	Age/ sex	Cancer/setting	Prior oxaliplatin exposure	Regimen	Index HSR: cycle/onset	Manifestations	Brown grade	HSR treatment
P01	65/F	Colon cancer; Adjuvant setting	No	FOLFOX (oxaliplatin, leucovorin, 5-FU)	Cycle 5; ~60 min	Skin: Erythema, pruritus, flushing, urticaria Cardiovascular: Tachycardia. Respiratory: Dyspnea, chest tightness	II	Diphenhydramine + methylprednisolone
P02	57/M	Colon cancer; Metastasis setting	Yes	FOLFOX (oxaliplatin, leucovorin, 5-FU) + bevacizumab	Cycle 7; 26 min	Skin: Erythema, pruritus, flushing, urticaria Cardiovascular: Tachycardia, hypertension Respiratory: Dyspnea, chest tightness. Gastrointestinal: Nausea, abdominal pain	II	Diphenhydramine + methylprednisolone
P03	69/M	Colon cancer; Metastasis setting	No	FOLFOX (oxaliplatin, leucovorin, 5-FU)	Cycle 2; ~60 min	Skin: Erythema, pruritus	I	Diphenhydramine
P04	71/M	Gastric cancer; Metastasis setting	Yes	FOLFOX (oxaliplatin, leucovorin, 5-FU) + Nivolumab	Cycle 8; ~210 min	Skin: Flushing Cardiovascular: Tachycardia, hypertension Respiratory: Dyspnea	II	Epinephrine + diphenhydramine + corticosteroids
P05	62/M	Colon cancer; Adjuvant setting	No	FOLFOX (oxaliplatin, leucovorin, 5-FU)	Cycle 7; 30 min	Skin: Erythema Gastrointestinal symptoms: Abdominal pain	II	Diphenhydramine
P06	76/M	Gastric cancer; Adjuvant setting	No	FOLFOX (oxaliplatin, leucovorin, 5-FU)	Cycle 2; ~60 min	Skin: Erythema, pruritus	I	Diphenhydramine + desloratadine
P07	52/M	Rectal cancer; Metastasis setting	Yes	FOLFOX (oxaliplatin, leucovorin, 5-FU) + bevacizumab (current course; historical FOLFOX HSR)	Cycle 11 (prior course); NR	Skin: Erythema, pruritus, flushing, urticaria Respiratory: Dyspnea, chest tightness Gastrointestinal: Nausea, vomiting, abdominal pain	III	NR
P08	54/F	Colon cancer; Metastasis setting	Yes	FOLFOX (oxaliplatin, leucovorin, 5-FU)	Cycle 2; NR	Skin: Erythema, pruritus. Respiratory: Dyspnea, chest tightness/	II	NR
P09	71/M	Gastric cancer; Metastasis setting	Yes	CAPOX (oxaliplatin + capecitabine)	Cycle 4; 7 5 min	Skin: Erythema, itching, flushing.	I	Methylprednisolone;
P10	54/M	Colon cancer; Metastasis setting	No	FOLFOX (oxaliplatin, leucovorin, 5-FU) + cetuximab	Cycle 7; ~ 60 min	Skin: Erythema, pruritus, flushing, urticaria Cardiovascular: Tachycardia Respiratory: Dyspnea	II	Epinephrine + diphenhydramine + methylprednisolone

### Characteristics of initial HSR

Index HSRs occurred after 2–11 preceding oxaliplatin cycles, with a median of six cycles (**Table 1**). Patient P07 underwent desensitization at the start of the current metastasis regimen because of a documented grade 3 HSR during cycle 11 of previous adjuvant oxaliplatin therapy. The index reactions were reassessed retrospectively according to their predominant clinical phenotype. All ten patients had cutaneous manifestations, including erythema, pruritus, or urticaria. Six patients also developed dyspnea or chest tightness; in all six, respiratory symptoms occurred with cutaneous manifestations rather than as isolated throat or respiratory complaints. Four patients had cardiovascular involvement, and three had gastrointestinal manifestations, including nausea or loose stools. No isolated cold-triggered syndrome suggestive of acute pharyngolaryngeal dysesthesia and no predominantly cytokine-release phenotype characterized by fever, chills, or rigors were identified. All index reactions were therefore categorized as type I-like clinical phenotypes, while recognizing that the underlying endotypes could not be confirmed. Three reactions were Brown grade 1, six were grade 2, and one was grade 3.

All index reactions resolved after interruption of the infusion and clinically indicated treatment. No patient underwent standard-rate oxaliplatin rechallenge before desensitization. Epinephrine was administered during the index reaction in two patients, whereas no epinephrine was required for any BTR during desensitization. The exact treatment of each index reaction is reported in **Table 1**. 

### Skin test results

All ten patients underwent skin prick testing followed by intradermal testing as part of the allergy and risk assessment; testing was not considered definitive confirmation or exclusion of oxaliplatin hypersensitivity. Skin prick testing was negative in all patients. Intradermal testing was positive in six patients, negative in three, and equivocal in one. The equivocal response consisted of a wheal increase of less than 1 mm and did not meet the prespecified criterion for positivity. Because sequential intradermal testing at higher concentrations was not performed, negative and equivocal findings were interpreted cautiously. All three patients with negative intradermal tests and the patient with the equivocal result initially underwent a four-bag procedure.

### Desensitization results

All ten patients underwent at least one oxaliplatin desensitization procedure, accounting for 48 procedures in total. Three patients initially received a three-bag procedure and seven received a four-bag procedure. Among the six patients with positive intradermal tests, three initially received three bags and three received four bags. The three patients with negative intradermal tests and the patient with the equivocal response received four bags. Two patients were subsequently de-escalated from four bags to three bags after at least three consecutively tolerated procedures. Because no prospectively defined allocation algorithm was used, these distributions should not be interpreted as comparisons between procedures (**Figure 1**).

**Figure 1:**
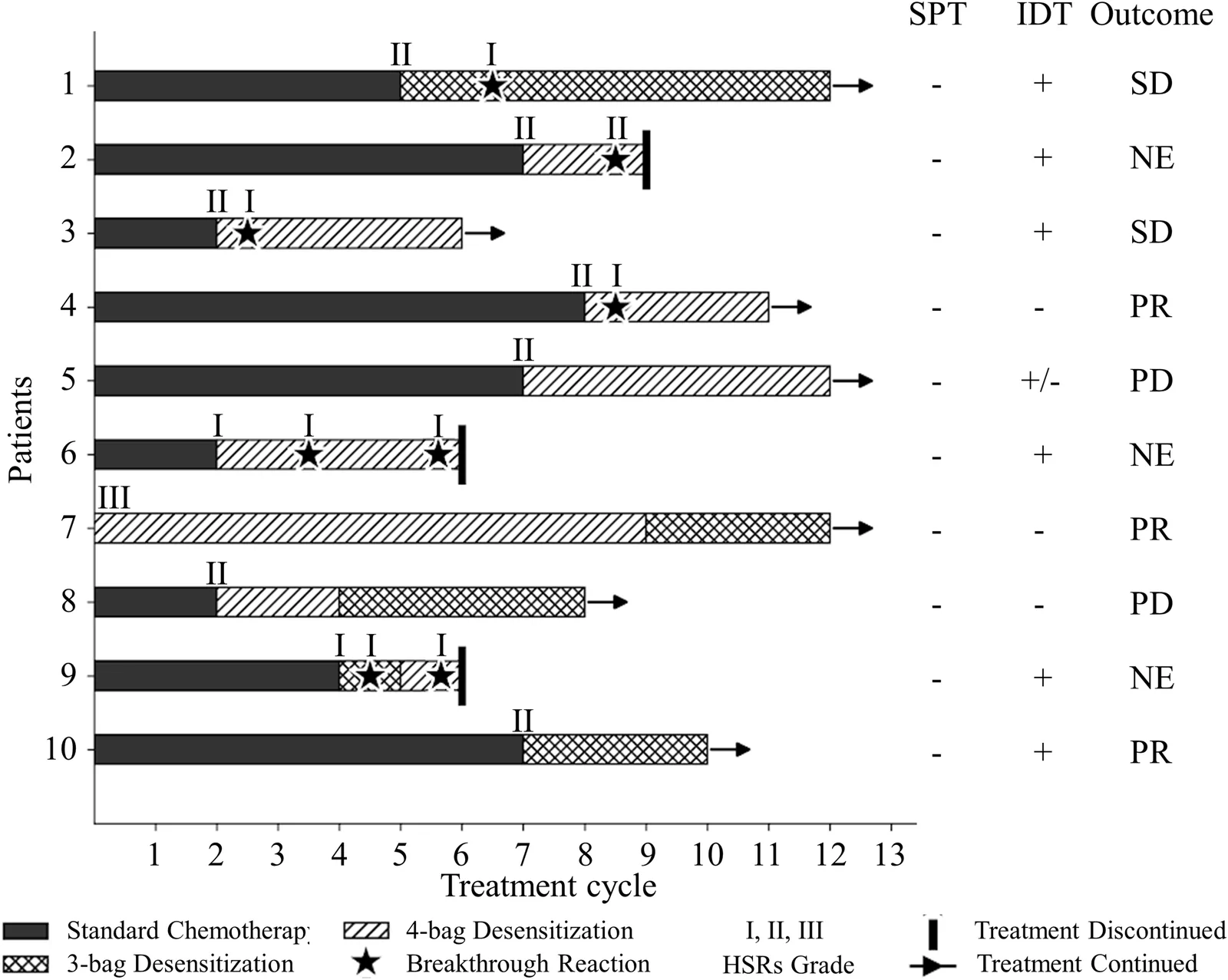
Longitudinal clinical course of oxaliplatin desensitization. The swimmer plot shows the individual treatment trajectories of ten patients. Solid bars denote standard oxaliplatin cycles administered before the index HSR. Hatched bars denote desensitization procedures using the three-bag or four-bag schedule, and stars indicate breakthrough reactions. Timeline endpoints indicate completion of planned treatment or permanent oxaliplatin discontinuation. Tumor responses are shown only as descriptive clinical context and should not be interpreted as outcomes caused by desensitization. SPT, skin prick test; IDT, intradermal test; PR, partial response; SD, stable disease; PD, progressive disease; NE, not evaluated.

Seven patients completed all additional oxaliplatin cycles planned by their oncology team and received a median of four additional cycles (interquartile range, 3–6). Eight BTRs occurred during 48 desensitization procedures, affecting six patients. Overall, 45 procedures (93.8%) were completed and three were terminated. Five of the eight procedures complicated by a BTR were completed after treatment and infusion resumption, whereas three were terminated. No BTR required epinephrine. Three patients did not continue oxaliplatin after BTRs, but the reasons were not uniform. P02 experienced a single BTR during the second desensitization procedure. The procedure was discontinued after the BTR, and the patient subsequently declined further oxaliplatin treatment in metastasis setting after discussion of potential benefits and risks. P06 discontinued oxaliplatin after recurrent BTRs despite sequential procedure modifications, including limitation of the maximum infusion rate and expansion from a 14-step to a more conservative 19-step procedure. P09 experienced a second BTR during the four-bag/17-step procedure. The infusion was not resumed, the procedure was terminated, and no further oxaliplatin was administered because the reaction had recurred despite escalation from the initial three-bag/12-step procedure. Five of the six patients with BTRs had positive intradermal tests; the remaining patient had a negative test. Event-level details are reported in **Supplementary Table S2**.

Among the seven patients who completed planned treatment, three had a partial response, two had stable disease, and two had progressive disease. These outcomes are presented descriptively and cannot be attributed to desensitization or additional oxaliplatin exposure.

## Discussion

Oxaliplatin remains an important component of systemic therapy for gastrointestinal malignancies, particularly colorectal and gastric cancers, in both adjuvant and metastasis settings (14–16). Oxaliplatin-associated HSRs can interrupt a clinically indicated regimen and prompt premature discontinuation. Although quality of life was not evaluated in our cohort, treatment interruption or unplanned regimen changes may increase patient burden. A retrospective comparison reported no significant difference in overall survival between oxaliplatin-hypersensitive patients treated through rapid drug desensitization and nonallergic controls receiving standard infusions, supporting the clinical importance of preserving access to indicated oxaliplatin therapy when appropriate (22). This case series describes the early implementation of a multidisciplinary desensitization pathway rather than a comparative evaluation of efficacy or safety.

Index HSRs occurred after a median of six oxaliplatin cycles, consistent with the pattern of reactions often emerging after repeated exposure (17, 18). Repeated prior exposure may be compatible with previous sensitization in some patients, but exposure history alone cannot establish an IgE-mediated mechanism or distinguish type I reactions from cytokine-release or mixed phenotypes. Most index reactions were Brown grade 1–2, and one was grade 3. Although all reactions had type I-like clinical features, their underlying endotypes could not be confirmed because tryptase, cytokine measurements, and BAT were unavailable.

Skin testing was used as one component of the allergy assessment rather than as definitive confirmation or exclusion of oxaliplatin hypersensitivity. Intradermal testing was positive in six patients, negative in three, and equivocal in one, whereas all skin prick tests were negative. Although the concentrations used were based on previously published oxaliplatin-specific protocols (10, 11), the use of a single intradermal concentration of 0.1 mg/mL may have reduced sensitivity and contributed to negative or equivocal findings. Accordingly, these results were interpreted together with the clinical history, and all patients with negative or equivocal intradermal tests underwent a four-bag procedure. Serum tryptase was unavailable, and BAT, which has been investigated as a potential biomarker of rapid desensitization outcomes in platinum allergy, was also not available in this cohort (21).

Five of the six patients who experienced a BTR had positive intradermal tests, while one of the three patients with a negative test also reacted during desensitization. The patient with an equivocal intradermal response did not experience a BTR. Given the cohort size, this pattern cannot establish predictive value or support a validated risk-stratification model. It is reported only as a descriptive observation and reinforces the need to interpret skin testing within the broader clinical assessment.

Seven of ten patients completed all additional oxaliplatin cycles planned by their oncology team. This proportion should not be interpreted as an efficacy estimate because the cohort was small, planned treatment duration varied, and treatment noncontinuation reflected both recurrent reactions and patient preference. Across 48 desensitization procedures, eight BTRs occurred in six patients. None required epinephrine. Large rapid drug desensitization series have reported high procedure-level completion rates (19, 20). Sloane et al. reported 2,177 desensitizations in 370 patients, with 93% associated with no or only mild reactions; Caiado et al. reported 1,471 procedures in 272 patients, with BTRs in 9.6% of infusions (23, 24). More recently, Torres Górriz et al. reported 570 platinum- or taxane-desensitization procedures in 120 patients, with the target dose achieved in 99.5% of procedures despite BTR rates of 24% for deferred rapid desensitization and 15% for same-day desensitization (25). Differences in patient selection, culprit agents, reaction phenotypes, desensitization protocols, and outcome definitions limit direct comparison with this small oxaliplatin-only institutional cohort. Two patients discontinued oxaliplatin after recurrent BTRs despite procedural modification: P06 after recurrence during a modified 19-step procedure and P09 after the second procedure was terminated because of a recurrent BTR despite escalation from a three-bag/12-step to a four-bag/17-step procedure. P02 declined further oxaliplatin after a single BTR in metastasis setting. Although the final BTRs preceding noncontinuation in P06 and P09 were Brown grade 1, both patients had recurrent reactions despite sequential procedure intensification; P02 had a single grade 2 BTR and declined further oxaliplatin after discussion of potential benefits and risks. Under the institutional decision framework described in Methods, noncontinuation was considered according to reaction recurrence, ability to complete the infusion, anticipated oncologic benefit, treatment alternatives, and patient preference rather than Brown grade alone. General desensitization guidance recommends individualized management of BTRs, and published reports describe further protocol modification or, in selected refractory cases, off-label omalizumab-supported desensitization; however, the evidence for omalizumab in this setting remains limited (26–28). These findings illustrate the distinction between procedure-level tolerance and patient-level treatment completion and do not support an unqualified conclusion regarding safety or efficacy.

The pathway evolved during the study period, and initial procedure allocation and subsequent modification were based on multidisciplinary clinical judgment rather than a prospectively defined algorithm. The study therefore does not compare the three-bag and four-bag procedures or validate the allocation strategy. Instead, it documents the early implementation of a pathway involving allergists, oncologists, clinical pharmacists, and oncology nurses and identifies areas requiring further standardization, including skin-testing procedures, protocol-allocation criteria, infusion calculations, and event-level documentation.

Seven of the ten patients in this cohort had colorectal cancer, which includes clinically and molecularly heterogeneous subgroups defined in part by hereditary predisposition, mismatch-repair status, and recurrent driver alterations (29–31). These features can inform treatment selection, and one recent retrospective study reported variation in chemotherapy-related adverse events according to Lynch syndrome mutation status (29). Because hereditary-cancer status and tumor molecular profiles were not systematically available in this series, their relationships with oxaliplatin HSRs or desensitization outcomes could not be assessed.

This study has several limitations. First, it was a retrospective, single-center case series of only ten patients, without a comparator group or inferential analysis. It therefore cannot establish the efficacy or comparative safety of desensitization, identify predictors of BTRs, or determine whether one procedure is superior to another. Second, the institutional pathway evolved during the study period, and decisions regarding initial procedure allocation, infusion resumption, subsequent modification, and treatment discontinuation were made case by case rather than according to a prospectively defined algorithm. Third, the cohort was heterogeneous with respect to cancer type, treatment setting, previous oxaliplatin exposure, prescribed dose, and number of additional treatment cycles. Fourth, skin testing used a skin prick concentration of 1 mg/mL followed by a single intradermal concentration of 0.1 mg/mL without sequential testing at higher concentrations, which may have reduced sensitivity and contributed to negative or equivocal results. Serum tryptase, cytokine measurements, and BAT were also unavailable, limiting confirmation of the underlying reaction endotypes. Fifth, the three-bag and four-bag procedures were individualized and were not applied uniformly across patients; the schedules presented in **Supplementary Tables S3** and **S4** are representative reconstructed examples rather than standardized protocols used identically throughout the cohort. Sixth, some preparation and infusion details required retrospective reconstruction, and exact reaction timing, cumulative dose at BTR onset, clinical manifestations, and vital-sign changes were not consistently available. Finally, patient preference contributed to treatment noncontinuation in one case, and patient-level treatment completion should therefore not be interpreted solely as a measure of desensitization success. These findings should be regarded as descriptive and hypothesis-generating.

In this early institutional case series, seven of ten patients completed all additional oxaliplatin cycles planned by their oncology team. However, eight BTRs occurred in six patients, and two patients discontinued oxaliplatin because of recurrent reactions despite procedural modification. The study documents the implementation of a multidisciplinary oxaliplatin desensitization pathway but does not establish efficacy, comparative safety, procedure superiority, or the predictive value of skin testing. Further protocol standardization and prospective evaluation are needed.

## Data Availability

The raw data supporting the conclusions of this article will be made available by the authors, without undue reservation.
