# Supplementary material for: Oxaliplatin desensitization for gastrointestinal cancer patients with hypersensitivity reactions: real-world experience from Vietnam
Source: Front Oncol. 2026 Sep 30;16:1876656. doi: 10.3389/fonc.2026.1876656 (PMC13661475; doi:10.3389/fonc.2026.1876656)
Supplement: Supplementary file 1 [file Table1.docx]

Supplementary Information

**Supplementary Table S1. Allergy evaluation, pathway allocation, and outcomes**

| **Patient ID** | **Time from index HSR to skin testing** | **SPT result** | **IDT result** | **Initial procedure 3-bag / 4-bag** | **Documented rationale for allocation** | **No. of RDD cycles** | **No. of BTR events** | **Patient-level outcome** | **Reason for permanent discontinuation** | **Best tumor response (descriptive)** |
| --- | --- | --- | --- | --- | --- | --- | --- | --- | --- | --- |
| P01 | 14 days | - | + | 3 bags and 16 steps | Case-by-case allocation; positive IDT | 7 | 1 | Completed planned oxaliplatin | NA | Stable disease |
| P02 | 38 days | - | + | 4 bags and 16 steps | More conservative pathway used; positive IDT | 2 | 1 | Permanently discontinued | BTR during RDD cycle 2 | Not evaluated |
| P03 | 15 days | - | + | 4 bags and 16 steps | More conservative pathway used; positive IDT | 4 | 1 | Completed planned oxaliplatin | NA | Stable disease |
| P04 | 13 days | - | - | 4 bags and 16 steps | Negative IDT and grade 2 index HSR; conservative pathway | 3 | 1 | Completed planned oxaliplatin | NA | Partial response |
| P05 | 15 days | - | +/- | 4 bags and 16 steps | Equivocal IDT; conservative pathway | 5 | 0 | Completed planned oxaliplatin | NA | Progressive disease |
| P06 | 14 days | - | + | 4 bags and 14 steps | More conservative pathway used; positive IDT | 4 | 2 | Permanently discontinued | Recurrent BTR despite protocol modification | Not evaluated |
| P07 | NR | - | - | 4 bags and 16 steps | Negative IDT and historical grade 3 HSR; conservative pathway | 12 | 0 | Completed planned oxaliplatin | NA | Partial response |
| P08 | NR | - | - | 4 bags and 15 steps | Negative IDT and grade 2 index HSR; conservative pathway. | 6 | 0 | Completed planned oxaliplatin | NA | Progressive disease |
| P09 | 26 days | - | + | 3 bags and 12 steps | Case-by-case allocation; positive IDT | 2 | 2 | Permanently discontinued | Recurrent BTR despite escalation | Not evaluated |
| P10 | NR | - | + | 3 bags and 12 steps | Case-by-case allocation; positive IDT | 3 | 0 | Completed planned oxaliplatin | NA | Partial response |

# Supplementary Table S2. Event-level characteristics and management of breakthrough reactions during oxaliplatin desensitization

| **Event ID** | **Patient ID** | **Desensitization cycle at which BTRs occur** | **Procedure 3-bag / 4-bag** | **Bag / step** | **Rate at onset (mL/h)** | **Brown grade** | **Immediate treatment medication** | **Epinephrine** | **Infusion resumed? step/rate** | **Subsequent protocol or treatment decision (premed/procedure)** | **Desensitization procedure outcome** |
| --- | --- | --- | --- | --- | --- | --- | --- | --- | --- | --- | --- |
| P01-BTR-01 | P01 | RDD cycle 2 | 3-bag/ 16 steps | 16 | 100ml/h | I | Methylprednisolon 40mg; diphenhydramine 20mg | No | Yes. 80ml/h | Omitted final high-rate step, maximum rate 80 ml/h | Completed |
| P02-BTR01 | P02 | RDD cycle 2 | 4-bag/16-step | 16 | 160ml/h | II | Methylprednisolon 40mg; diphenhydramine 20mg | No | No | NA | Discontinued (patient declined) |
| P03-BTR01 | P03 | RDD cycle 1 | 4-bag/16-step | 16 | 160ml/h | I | Methylprednisolon 40mg; diphenhydramine 20mg | No | Yes. 80ml/h | Omitted final high-rate step, maximum rate 80 ml/h | Completed |
| P04-BTR01 | P04 | RDD cycle 1 | 4-bag/16-step | 16 | 160ml/h | I | Methylprednisolon 40mg; diphenhydramine 20mg | No | Yes | Increase premedication (dexamethasone 12mg -> 20mg) | Completed |
| P06-BTR01 | P06 | RDD cycle 2 | 4-bag/14 steps | 14 | 160ml/h | I | Methylprednisolon 40mg; diphenhydramine 20mg | No | Yes. 80ml/h | Change the desensitization procedure to 4 bags/19 steps, maximum rate 80ml/h |  |
| P06-BTR02 | P06 | RDD cycle 4 | 4-bag/19 steps | 19 | 80ml/h | I | Methylprednisolon 40mg; diphenhydramine 20mg | No | No | No further desensitization | Discontinued due to recurrent BTR |
| P09-BTR01 | P09 | RDD cycle 1 | 3-bag/12 steps | 12 | 160ml/h | I | Methylprednisolon 40mg; diphenhydramine 20mg | No | Yes. 80ml/h | Escalated to 4-bag/17-step |  |
| P09-BTR02 | P09 | RDD cycle 2 | 4-bag/17 steps | 17 | 160ml/h | I | Methylprednisolon 40mg; diphenhydramine 20mg | No | No | No further desensitization | Discontinued due to recurrent BTR |

# Supplementary Table S3. Representative three-bag oxaliplatin desensitization procedure

**Prescribed oxaliplatin dose: 150 mg**

| **Step** | **Solution** | **Infusion rate (ml/h)** | **Time (min)** | **Volume infused per step (ml)** | **Dose per step (mg)** | **Cumulative dose (mg)** |
| --- | --- | --- | --- | --- | --- | --- |
| **Solution 1:** Mix 5ml of solution 2 into 45ml of 5% glucose solution (concentration: 0.003mg/ml)  **Prepared solution:** 0.0564mg/18.8ml  **Administration:** 18.8ml, infuse the entire bottle | | | | | | |
| 1 | 1 | 5 | 15 | 1.3 | 0.00375 | 0.00375 |
| 2 | 1 | 10 | 15 | 2.5 | 0.0075 | 0.01125 |
| 3 | 1 | 20 | 15 | 5.0 | 0.015 | 0.02625 |
| 4 | 1 | 40 | 15 | 10.0 | 0.03 | 0.05625 |
| Total infusion of solution 1 | | | 60 | 18.8 | 0.05625 |  |
| **Solution 2:** Mix 5ml of solution 3 into 45ml of 5% glucose solution (concentration: 0.03mg/ml)  **Prepared solution:** 1.125mg/37.5ml  **Administration:** 37.5ml, infuse the entire bottle | | | | | | |
| 5 | 2 | 10 | 15 | 2.5 | 0.075 | 0.13125 |
| 6 | 2 | 20 | 15 | 5.0 | 0.15 | 0.28125 |
| 7 | 2 | 40 | 15 | 10.0 | 0.3 | 0.58125 |
| 8 | 2 | 80 | 15 | 20.0 | 0.6 | 1.18125 |
| Total infusion of solution 2 | | | 60 | 37.5 | 1.125 |  |
| **Solution 3:** 150mg oxaliplatin diluted into 500ml of 5% glucose solution (concentration: 0.3mg/ml)  **Prepared solution:** 148.5mg/495ml  **Administration:** 495ml, infuse the entire bottle | | | | | | |
| 9 | 3 | 20 | 15 | 5.0 | 1.5 | 2.68125 |
| 10 | 3 | 40 | 15 | 10.0 | 3.0 | 5.68125 |
| 11 | 3 | 80 | 15 | 20.0 | 6.0 | 11.68125 |
| 12 | 3 | 160 | 172.5 | 460 | 138 | 149.68125 |
| Total infusion of solution 3 | | | 217.5 | 495 | 148.5 |  |

Total time (min) = 337.5 = 5.625h

# Supplementary Table S4. Representative four-bag oxaliplatin desensitization procedure

**Prescribed oxaliplatin dose: 140 mg**

| **Step** | **Solution** | **Infusion rate (ml/h)** | **Time (min)** | **Volume infused per step (ml)** | **Dose per step (mg)** | **Cumulative dose (mg)** |
| --- | --- | --- | --- | --- | --- | --- |
| **Solution 1:** Mix 5ml of solution 2 into 45ml of 5% glucose solution (concentration: 0.00028mg/ml)  **Prepared solution:** 0.005264mg/18.8ml  **Administration:** 18.8ml | | | | | | |
| 1 | 1 | 5 | 15 | 1.3 | 0.0004 | 0.0004 |
| 2 | 1 | 10 | 15 | 2.5 | 0.0007 | 0.0011 |
| 3 | 1 | 20 | 15 | 5.0 | 0.0014 | 0.0025 |
| 4 | 1 | 40 | 15 | 10.0 | 0.0028 | 0.0053 |
| Total infusion of solution 1 | | 60 | 18.8 | 0.0053 |  | |
| **Solution 2:** Mix 5ml of solution 3 into 45ml of 5% glucose solution (concentration: 0.0028mg/ml)  **Prepared solution:** 0.05264mg/18.8ml  **Administration:** 18.8ml | | | | | | |
| 5 | 2 | 5 | 15 | 1.3 | 0.0035 | 0.0088 |
| 6 | 2 | 10 | 15 | 2.5 | 0.0070 | 0.0158 |
| 7 | 2 | 20 | 15 | 5.0 | 0.0140 | 0.0298 |
| 8 | 2 | 40 | 15 | 10.0 | 0.0280 | 0.0578 |
| Total infusion of solution 2 | | 60 | 18.8 | 0.0525 |  | |
| **Solution 3:** Mix 5ml of solution 4 into 45ml of 5% glucose solution (concentration: 0.028mg/ml)  **Prepared solution:** 1.05mg/37.5ml  **Administration:** 37.5ml | | | | | | |
| 9 | 3 | 10 | 15 | 2.5 | 0.0700 | 0.1278 |
| 10 | 3 | 20 | 15 | 5.0 | 0.1400 | 0.2678 |
| 11 | 3 | 40 | 15 | 10.0 | 0.2800 | 0.5478 |
| 12 | 3 | 80 | 15 | 20.0 | 0.5600 | 1.1078 |
| Total infusion of solution 3 | | 60 | 37.5 | 1.0500 |  | |
| **Solution 4:** 140mg oxaliplatin diluted into 500ml of 5% glucose solution (concentration: 0.28mg/ml)  **Prepared solution:** 138.6mg/495ml  **Administration:** 495ml | | | | | | |
| 13 | 4 | 20 | 15 | 5.0 | 1.4000 | 2.5078 |
| 14 | 4 | 40 | 15 | 10.0 | 2.8000 | 5.3078 |
| 15 | 4 | 80 | 15 | 20.0 | 5.6000 | 10.9078 |
| 16 | 4 | 160 | 172.5 | 460 | 128.8 | 139.7078 |
| Total infusion of solution 4 | | 217.5 | 495 | 138.6 |  | |

Total time (min) = 397.5 = 6.625h

# Abbreviations and coding rules

| **Field / abbreviation** | **Definition / permitted values** |
| --- | --- |
| BTR | Breakthrough reaction occurring during a desensitization procedure. |
| RDD | Rapid drug desensitization procedure. |
| SPT / IDT | Skin prick test / intradermal test. |
| NR | Not recorded in the source medical record. |
| NA | Not applicable. |
| Not performed | Investigation or intervention was explicitly not performed. |
| Procedure completion | The intended oxaliplatin dose was administered during that procedure, with or without a managed BTR. |
| Patient-level completion | All oxaliplatin cycles planned by the treating oncology team were completed without permanent discontinuation due to BTR. |
| Tumor response | Descriptive clinical context only; do not attribute causally to desensitization. |
